# Heat Shock Protein 72 Expressing Stress in Sepsis: Unbridgeable Gap between Animal and Human Studies—A Hypothetical “Comparative” Study

**DOI:** 10.1155/2014/101023

**Published:** 2014-01-12

**Authors:** George Briassoulis, Efrossini Briassouli, Diana-Michaela Fitrolaki, Ioanna Plati, Kleovoulos Apostolou, Theonymfi Tavladaki, Anna-Maria Spanaki

**Affiliations:** ^1^Pediatric Intensive Care Unit, University Hospital, School of Health Sciences, University of Crete, Voutes Area, 71110 Heraklion, Crete, Greece; ^2^1st Department of Propaedeutic Internal Medicine, Laiko, University General Hospital, University of Athens, 17 Agiou Thoma, 115 27 Athens, Greece; ^3^Department of Clinical Chemistry, School of Medicine, University of Crete, Voutes Area, 71110 Heraklion, Crete, Greece; ^4^National and Kapodistrian University of Athens, First Critical Care Department, Evaggelismos Hospital, Ipsilantou 45, 10676, Athens, Greece

## Abstract

Heat shock protein 72 (Hsp72) exhibits a protective role during times of increased risk of pathogenic challenge and/or tissue damage. The aim of the study was to ascertain Hsp72 protective effect differences between animal and human studies in sepsis using a hypothetical “comparative study” model. Forty-one in vivo (56.1%), in vitro (17.1%), or combined (26.8%) animal and 14 in vivo (2) or in vitro (12) human Hsp72 studies (*P* < 0.0001) were enrolled in the analysis. Of the 14 human studies, 50% showed a protective Hsp72 effect compared to 95.8% protection shown in septic animal studies (*P* < 0.0001). Only human studies reported Hsp72-associated mortality (21.4%) or infection (7.1%) or reported results (14.3%) to be nonprotective (*P* < 0.001). In animal models, any Hsp72 induction method tried increased intracellular Hsp72 (100%), compared to 57.1% of human studies (*P* < 0.02), reduced proinflammatory cytokines (28/29), and enhanced survival (18/18). Animal studies show a clear Hsp72 protective effect in sepsis. Human studies are inconclusive, showing either protection or a possible relation to mortality and infections. This might be due to the fact that using evermore purified target cell populations in animal models, a lot of clinical information regarding the net response that occurs in sepsis is missing.

## 1. Introduction

Sepsis is an inflammation-induced syndrome resulting from a complex interaction between host and infectious agents.  It is considered severe when associated with acute organ dysfunction, which accounts for the main cause underlying sepsis-induced death. Despite increasing evidence in support of antioxidant [1], anti-inflammatory [2], or immune-enhancing [3] therapies in sepsis, recent studies failed to establish a correlation between antiseptic pathway-based therapies and improvement of sepsis [4] or septic shock [5] or among immune-competent patients [6].

Rapid expression of the survival gene heat shock protein 72 (Hsp72) was shown to be critical for mounting cytoprotection against severe cellular stress, like elevated temperature [7]. Intracellular Hsps are upregulated in cells subjected to stressful stimuli, including inflammation and oxidative stress exerting a protective effect against hypoxia, excess oxygen radicals, endotoxin, infections, and fever [8]. Recent studies imply that different biological disease processes and/or simple interventions may interfere with high temperature stress, leading to different clinical outcome in patients with and without sepsis [9]. In septic patients, administration of antipyretics independently associated with 28-day mortality, without association of fever with mortality [9]. Importantly, fever control using external cooling was safe and decreased vasopressor requirements and early mortality in septic shock [10].

Inducible Hsp72 is also found extracellularly where it exhibits a protective role by facilitating immunological responses during times of increased risk of pathogenic challenge and/or tissue damage [11]. Experimental data provide important insights into the anti-inflammatory mechanisms of stress proteins protection and may lead to the development of a novel strategy for treatment of infectious and inflammatory disorders [12]. However, although overexpression of stress proteins signals danger to inflammatory cells and aids in immune surveillance by transporting intracellular peptides to immune cells [13], it has also been linked to a deleterious role in some diseases [14]. In addition, serum Hsp72 levels were shown to be modulated according to the patient oxidant status whereas increased serum Hsp72 was associated with mortality in sepsis [15].

The purpose of this basic research-related review in critical care is to document the available evidence on the role of Hsp72 in sepsis, reporting both the state of the art and the future research directions. It might be that potential therapeutic use of stress proteins in prevention of common stress-related diseases involves achieving optimal balance between protective and immunogenic effects of these molecules [16]. In this review, we will attempt to classify experimental and clinical studies on Hsp72 in sepsis and to compare their results on inflammation, organ function, and outcome; we will also briefly discuss the mechanisms on how stress proteins might exert their protective or negative role in the disease development and highlight the potential clinic translation in the research field.

## 2. Materials and Methods

Human or animal in vivo or in vitro studies examining the beneficial effect of intra- or extracellular Hsp72 expression in sepsis were included in this study. The PRISMA [17] search method for identification of studies consisted of searches of PubMed database (1992 to September 2012) and a manual review of reference lists using the search term: “Hsp70 or 72.” The search output was limited with the search filter for any of: sepsis; severe sepsis; bacterial lipopolysaccharide (LPS); endotoxin. References in selected studies were examined also. The title and abstract of all studies identified by the above search strategy were screened, and the full text for all potentially relevant studies published in English was obtained. The full text of any potentially relevant studies was assessed by five authors (DMF, EB, IP, AK, and TT). The same authors extracted data from the published studies.

### 2.1. Statistical Analysis

Proportions of methods used and results findings were compared by the *χ* ^2^ test. A two-sided alpha of 0.05 was used for statistical significance. The results were analyzed using SPSS software (version 20.0, SPSS, Chicago, IL, USA).

## 3. Results

Our search identified 411 PubMed titles and abstracts. After excluding duplicates, studies with no original data, or data insufficient to evaluate or those whose outcome was ischemia/reperfusion injury or others, 55 articles were finally included for analysis. The aim of this minireview was not to examine the quality of studies, but to describe induction methods and to compare in vivo and in vitro methods and results regarding a potential protective role for Hsp72 in human and animal sepsis.

### 3.1. Animals

Forty-one in vivo (23, 56.1%), in vitro (7, 17.1%), or combined (11, 26.8%) animal studies fulfilling the research criteria regarding the role of Hsp72 in sepsis were enrolled in analysis (Tables 1(a), 1(b), and 1(c)). In only 6 studies transgenic animals (4Hsp^−/−^ (9.8%), 2 overexpressing the human Hspa12b gene (4.9%)) were used (14.6%), all in mice (*P* < 0.03). Hsp72 induction methods used in rats differed from those used in mice (*P* < 0.0001). Hsp72 induction was attempted most often using heat shock (rats 9, 37.5%; mice 2, 12.5%), glutamine (Gln) (rats 7, 29.2%; mice 4, 25%; sheep 1, 100%), or combined Gln with additional inducer (rats 1, 4.2%; mice 2, 12.6%). In 7 rats Hsp72 was induced through adenoviral vector Hsp72 (AdHSP) (3, 12.5% of studies in rats) or various recombinant Hsp72 (rHsp72) preparations (4, 16.7%) compared to 3 mice studies where AdHSP, bovine rHsp72 preconditioning, or overexpressed Hsp72 within the intestinal epithelium was used (6.2%). Hsp72 gene-transfected models (3, 18.8%) or cecal ligation and puncture (CLP) with LPS or injection of microorganisms (2, 12.5%) were used only in mice studies.

**Figure 1:**
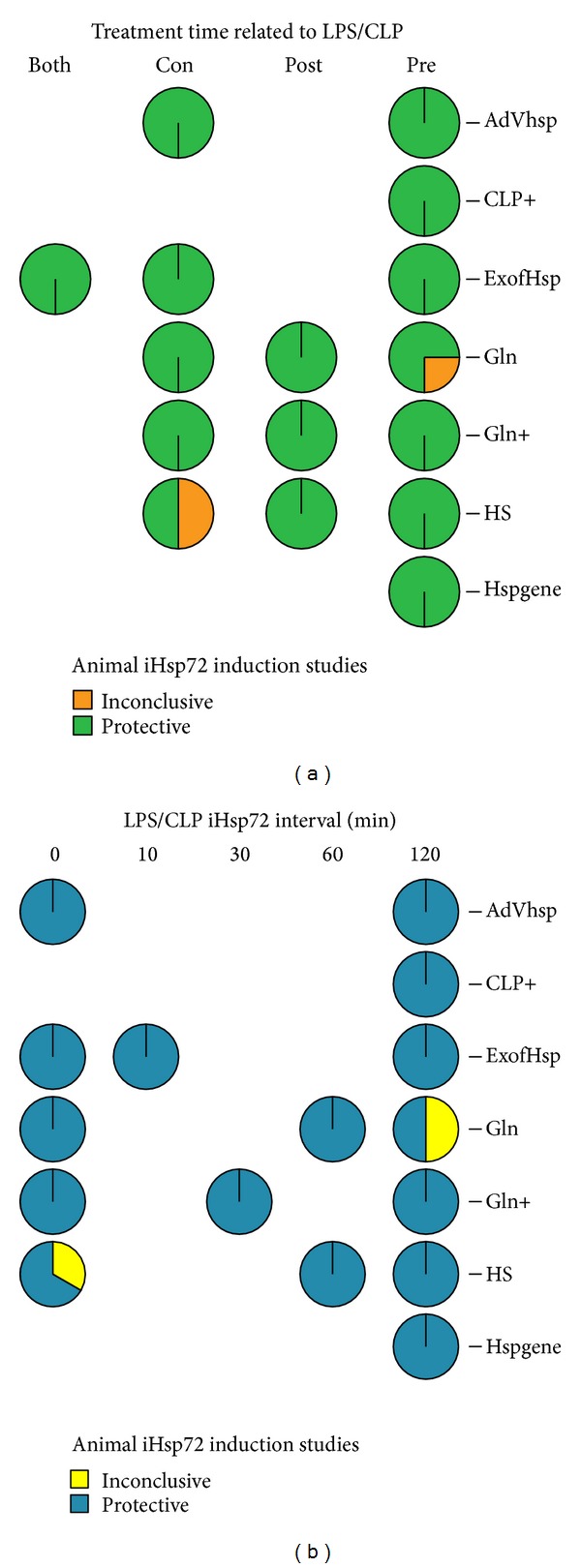
(a) Preventive effect was achieved by all induction methods used irrespective of the challenge period or (b) time lapse between the sepsis insult and the Hsp72 induction: LPS, bacterial lipopolysaccharide; CLP, caecal ligation and puncture; iHsp72, inducible heat shock protein 72; Pre, pre-treatment; Post, posttreatment; both, trials with pre- and postexperiments; Con, concomitant; AdHSP, adenoviral vector Hsp72; exogHsp, exogenous Hsp72 preparations; Gln, glutamine; +, additional challenge; HS, heat stress; Hspgene, Hsp72 gene-transfected models.

In more than half of the studies induction was attempted in a pretreatment mode (10, 62.5% for mice; 13, 54.2% for rats induction after LPS injection or CLP), followed by a concomitant mode in rats (6, 25%) or a posttreatment one in mice (4, 25%). The different time intervals used before or after experimental sepsis, most often 1-2 hours, did not differ among groups. Preventive effect was achieved by most induction methods used in mice or rats (39/41, 95.1%), irrespective of the challenge period or timing used (Figures 1(a) and 1(b)). Two studies, one carried out in sheep and one in rats, were inconclusive. In all septic animal models, any Hsp72 induction method tried increased intracellular Hsp72 (41/41, 100%), reduced proinflammatory cytokines (28/29 studies involving cytokine measurements), organ damage (27/27), clinical deterioration (19/20), and enhanced survival (18/18).

### 3.2. Patients

Only 14 human in vivo (2) and in vitro (12) Hsp72 studies were identified (Tables 2(a) and 2(b)): human peripheral blood mononuclear cells (hPBMC) 9 studies, 64.3%; polymorphonuclear leukocytes (hPMNL) 2 studies, 14.3%; lymphocytes (hPBLC) 1 study, 7.1%; in vivo (children or adults' serum levels) 2 studies, 14.3%. Of those, hPBMC were used in only 2 studies with septic patients but in 6 with healthy volunteers. Heat stress (HS) or acclimation was used in 5 studies (35.7%), Gln administration in 2 in association with LPS (14.3%), recombinant human Hsp72 in 1 (7.1%), and either inhibitor or agonist in 1 (7.1%). In 4 studies no challenge or only LPS (28.6%) was used. In only 1 out of 6 (16.7%) studies in septic patients induction Hsp72 methods were attempted compared to 100% in the studies with healthy (7) or ARDS (1) patients (*P* < 0.006). Protection markers studied were apoptosis (3 studies, 21.3%), HS (2 studies, 14.3%), oxidative damage, hospital infections, hemodynamic instability, and ARDS (1 study each, 7.1%).

Intracellular Hsp72 was induced in 8 in vitro studies (57.1%, 6 in healthy, 2 in septic) and inhibited in 3 (21.4%, 2 in septic, 1 in ARDS patients). Of the 6 studies in septic patients, intracellular Hsp72 was increased in 2 (33%), inhibited in 2 (33%), and not measured in 2. With the exception of sodium arsenite, neither Gln nor HS were tested in these studies. Extracellular Hsp72, measured in 1 in vitro and in 2 in vivo studies, was shown to increase in sepsis, especially in septic shock or in those who died (14.3% of human studies).

**Figure 2:**
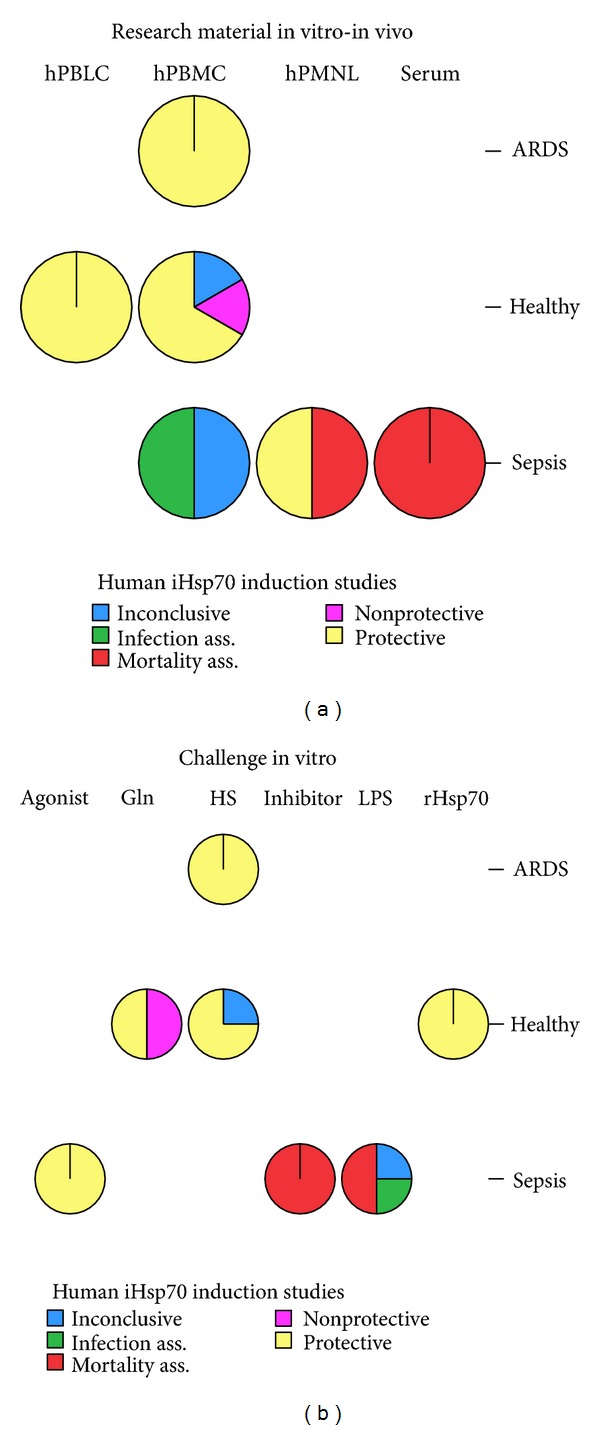
(a) Increased serum Hsp72 in septic patients was associated with mortality whereas human cell studies with Hsp72 induction were either inconclusive or protective or even partially associated with mortality and infection; (b) heat pretreatment and/or glutamine incubation and recombinant or Hsp72 agonists (sodium arsenite) partially protected human cells compared to the nonchallenged human cells or to those challenged with Hsp72 inhibitors (quercetin) or LPS alone (*P* < 0.04). Positive Hsp72 induction human in vitro studies were tried in healthy individuals or ARDS patients compared with 1 study in septic patients' cells (*P* < 0.02) whereas negative human Hsp72 studies (LPS, quercetin) or neutral studies (no induction) were only examined in septic human cells: iHsp72, inducible heat shock protein 72; hPBMC, human peripheral blood mononuclear cells; hPMNL, human peripheral polymorphonuclear leukocytes; hPBMC, human peripheral blood lymphocytes; ARDS, acute respiratory distress syndrome; Gln, glutamine; HS, heat stress; LPS, bacterial lipopolysaccharide; rHsp72, recombinant Hsp72.

Increased intracellular Hsp72 was protective in half of the human studies (50%); regarding the 9 positive (HS, Gln, exogenous Hsp72) in vitro induction Hsp72 human studies 7 (77.8%) were protective (Figure 2(a)) and 2 inconclusive (11.1%) or nonprotective (11.1%). Of the induction methods used, protection offered HS (4/5, 80%), glutamine (1/2, 50%), rHsp72 and sodium arsenite (1/1, 100% each) (Figure 2(b)). In contrast, of the 2 in vivo (serum Hsp72 measurements), 2 in vitro endotoxin induced (LPS or CLP), and 1 Hsp72 inhibitor human studies, none was shown to be associated with a better outcome (*P* < 0.02); 3 studies were associated with mortality (60%) and 1 with infection (20%) or were inconclusive (20%). Septic patients' studies were positive for protection in only 1 out of 6 (16.7%) compared to 5 out of 7 (71.4%) in healthy and 100% in ARDS patients (*P* < 0.06).

### 3.3. Human Compared to Animal Studies

**Figure 3:**
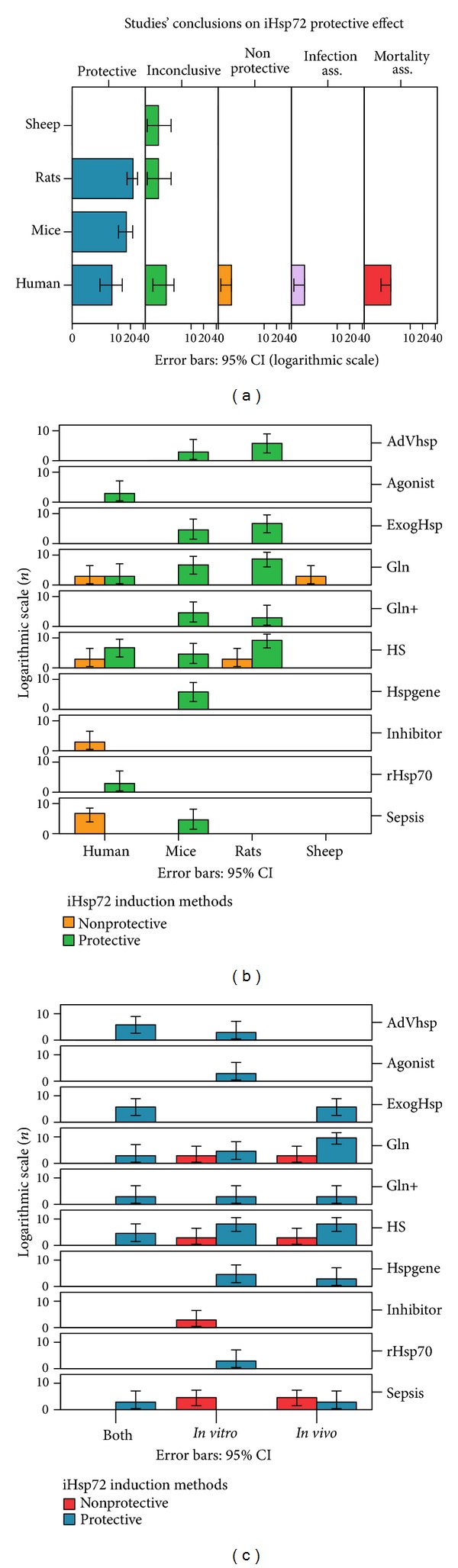
(a) Diagram showing summaries of conclusions regarding the Hsp72 protective effects in sepsis in human and animal studies (*P* < 0.008); (b) human Hsp72 induction methods showed inconsistent results compared to the unanimous Hsp72 protective results in experimental sepsis with any attempted induction method; selection of any induction method, however, did not affect results; (c) Hsp72 induction protective effect using various induction methods was not influenced by the in vitro, in vivo, or combined study method selected: iHsp72, inducible heat shock protein 72; AdHSP, adenoviral vector Hsp72; exogHsp, exogenous Hsp72 preparations; Gln, glutamine; +, additional challenge; HS, heat stress; rHsp72, recombinant Hsp72; Hspgene, Hsp72 gene-transfected models; both, in vitro and in vivo experiments.

Out of a total of 55 enrolled studies, only 2 in vivo human studies (3.6%) have been reported on the role of Hsp72 in sepsis compared to 7 mice (12.7%) and 15 rat (27.3%) in vivo studies (*P* < 0.0001); in contrast 12 human (21.8%) studies have been reported in vitro compared to only 2 in rats (3.6%) and 5 in mice (9.1%); 4 mice (7.3%) and 7 rat (12.7%) combined in vitro-in vivo studies have also been reported. Of the 14 human studies, 50% showed a protective Hsp72 effect compared to 95.8% protection shown in animal studies (Figure 3(a)). When restricted to the septic patients' studies, however, only 1 out of 6 (16.7%) demonstrated an Hsp72 protective effect compared to 95.8% protection shown in animal studies (*P* < 0.0001). In addition, only human studies reported Hsp72-associated mortality (21.4%) or infection (7.1%) or reported results (14.3%) to be nonprotective (*P* < 0.001).

Most of the human studies were prospective observational experimental controlled studies (57.1%) and only 1 randomized study (7.1%) compared to prospective controlled animal studies (100%, *P* < 0.0001). All other human studies were experimental control (14.3%) or noncontrolled (14.3%) studies. Induction methods used differed significantly (*P* < 0.02), increasing Hsp72 in 57.1% of the human as compared to 100% of animal studies (*P* < 0.02). Only 6 (42.9%) human studies included septic patients compared to 41 (100% experimental sepsis) in animal studies (*P* < 0.0001). Although differed among Hsp72 study populations (*P* < 0.001) or methodology selected (*P* < 0.02), the various induction methods used did not affect the Hsp72 offered protection (Figures 3(b) and 3(c)).

## 4. Discussion

Hsps70 are emerging as powerful dichotomous immune-modulatory molecules that can have stimulatory and inhibitory effects on immune responses [63]. In our hypothetical “comparative study” model, we found that the balance between Hsp72 promotion and control of inflammatory responses and sepsis outcome differed unpredictably between human and animal studies. Clinical studies were inconclusive, showing either a low probability of protection (16.7% among septic patients) or even a possible relation to mortality and infections. In contrast, almost all (94.7%) septic animal in vivo and in vitro studies showed a biochemical, biological, and clinical protective effect for Hsp72 in sepsis. This might be due to the fact that using evermore purified target cell populations to provide insight into the direct effects of molecules on cells, a lot of clinical information regarding the net response that occurs in vivo is missing [63].

### 4.1. Stress Proteins Induction

Sepsis, endotoxin tolerance, and heat shock all display downregulation of innate immunity, sharing a common immune suppressive effect, possibly through HS factor 1 (HSF1) mediated competitive inhibition of nuclear factor kappa-B (NF-*κ*B) binding [45]. It has been shown that multiple chaperones or cochaperones, including Hsp72, tend to form a complex with HSF1 monomers [64]. Once a cell is exposed to stress, these chaperones and cochaperones bind to denatured and damaged proteins, thereby “releasing” the nonactive HSF1 monomers to subsequently undergo homotrimerization [65]. However, while homotrimerization is sufficient for DNA binding and nuclear translocation, the magnitude and duration of transcriptional activity are regulated by inducible phosphorylation of specific serine residues of HSF1 by several protein kinases (Erk1/2, glycogen synthase kinase, protein kinase C) [64].

Once inside the nucleus, HSF1 binds to a heat shock element (HSE) in the promoter of  Hsp genes, which is defined by a tandem repeat of the pentamer nGAAn arranged in an alternating orientation either “head to head” (e.g., 5′-nGAAnnTTCn-3′) or “tail to tail” (e.g., 5′-nTTCnnGAAn-3′) [66], resulting in the upregulation of stress protein gene expression [67]. Thus, the intracellular accumulation of denatured or improperly folded proteins in response to stress is believed to be the universal signal resulting in the stress-induced gene expression of stress proteins [68, 69] which is proportional to the severity of the stress [70]. Besides the innate immune response stress proteins seem to activate also the adaptive immune response [71]. Thus, they have the capacity to elicit a pathogen-specific immune response [72] and to mediate the induction of peptide-specific immunity, eliciting potent T cell responses against the chaperoned peptide [73].

### 4.2. Experimental Hsp72 Studies

Hsp72 is the most highly induced stress protein in cells and tissues undergoing the stress response [74] and is central to the cytoprotective properties in patients with a variety of critical illnesses [52] or injuries [75]. Cell cycle components, regulatory proteins, and proteins in the mitogenic signal cascade may be protected by the molecular chaperone Hsp72 during periods of stress, by impairing proteasomal degradation of IkappaBalpha (I*κ*Ba) [47]. In addition, binding of Hsp72 to the Ser/Thr protein kinase IRE1a enhances the IRE1a/X-box binding protein XBP1 signaling at the endoplasmic reticulum and inhibits endoplasmic reticulum stress-induced apoptosis [76]. Thus, increased expression of Hsp72 by gene transfer/transfection has been demonstrated to confer protection against in vitro toxicity secondary to lethal hyperthermia [77], endotoxin [78], nitric oxide [79], hyperoxia [80], lung inflammation and injury [81], and in vivo ischemia-reperfusion injury [82]. On the contrary, microinjection of anti-Hsp72 antibody into cells impaired their ability to achieve thermotolerance [83].

We showed that in septic animal models, all reported Hsp72 induction methods increased intracellular Hsp72; this was associated with reduced proinflammatory cytokines, decreased organ damage, clinical improvement, and enhanced survival. Analysis of reviewed studies showed differed methodology approaching the Hsp72 biological and/or genetic implication in the sepsis process.

#### 4.2.1. Transgenic Animals

When challenged with systemic endotoxin, HSF1-deficient [84] or *Hsp722* ^−/−^ mice [49] had increased apoptosis and mortality compared to wild-type (*WT*) mice. Hsp72 expression was also required for Gln's protective effects on survival and tissue injury [34], an effect not seen in *Hsp72* ^−/−^ mice [85]. On the contrary, using transgenic mice overexpressing the human Hspa12b gene, Hsp72 attenuated the endotoxin-induced cardiac dysfunction and leucocyte infiltration into the myocardium [51].

#### 4.2.2. Hsp72 Overexpression with Adenovirus Injection (AdHSP)

Hsp72 overexpression with adenovirus injection prevented the LPS-induced increase in tumor necrosis factor-alpha (TNF*α*) and IL-6 levels associated with inhibited I*κ*B*α* degradation [36] through NF-*κ*B pathway [47]. Increases in levels of Hsp72 by gene transfection attenuated LPS- or TNF*α*-induced high mobility group box protein-1 (HMGB1) cytoplasmic translocation and release [12], decreased inducible NO synthase (iNOS) messenger RNA expression [45], and protected cells from programmed cell death [46]. Thus, AdHSP protected against sepsis-induced lung injury [86] by reducing nuclear caspase-3 [87], prevented alveolar type II cell proliferation [88], and improved short-term survival following CLP [89].

#### 4.2.3. Exogenous Hsp72

At the cellular level, Hsp72 preparations not only inhibited LPS-induced reactive oxygen species production and decreased NO expression in macrophages, but they also partially normalized the disturbed neutrophil apoptosis [37]. Prophylactic administration of exogenous human Hsp72 normalized inflammatory responses [38], limited host tissue damage [48], and reduced mortality rates [39]. Liposomal transfer of Hsp72 into the myocardium abolished LPS-induced contractile dysfunction [44], reduced mortality rates, and modified hemostasis and hemodynamics [40]. Intestinal Hsp72 overexpression reversed toll-like receptor (TLR)-4-induced cytokines and enterocyte apoptosis and prevented and treated experimental necrotizing enterocolitis [50]. Thus, mammalian Hsp72 appears to be an attractive target in therapeutic strategies designed to stimulate endogenous protective mechanisms against many deleterious consequences of septic shock by accelerating the functional recovery of susceptible organs in humans [40, 90].

#### 4.2.4. Glutamine

Although Gln has little effect under basal conditions [43], endotoxin-treated animals given Gln exhibited dramatic increases in tissue Hsp72 expression [26], marked reduction of end-organ damage [28], attenuation of cytokine release [41] and peroxide biosynthesis, and improved vascular reactivity [29] associated with a significant decrease in mortality [91]. The molecular mechanism of Gln-induced Hsp72 expression appears to be mediated via enhancement of O-linked *β*-N-acetylglucosamine modification and subsequently to increased levels of endonuclear HSF1 expression [43] and HSF1 transcription activity [42].

In a recent study, septic mice with Gln administration showed less severe damage to the kidneys and exhibited decreased HMGB1 and TLR4 in kidney tissues [35]. In Gln-treated rats, lung Hsp72 and HSF1-p expressions were enhanced [32, 92], lung HMGB1 expression and NF-*κ*B DNA-binding activity were suppressed, and ARDS was attenuated and survival improved [33]. By inducing Hsp72, Gln attenuated LPS-induced cardiomyocyte damage [42] and left ventricular dysfunction [27] whereas Gln-treated sheep had a greater increase in myocardial Hsp72 immunoreactivity without aggravating the hyperdynamic circulation after endotoxemia [31]. In a rat brain model of endotoxemia, Gln upregulated the expression of Hsp72 and decreased the magnitude of apoptosis by inhibiting the translocation of NF-*κ*B from the cytoplasm to the nucleus [30].

#### 4.2.5. Hyperthermic Heat Shock

Subjected to a brief hyperthermic heat shock, Hsp72 conferred protection against sepsis-related circulatory fatality via inhibition of iNOS gene expression through prevention of NF-*κ*B activation in cellular processes that included prevention of I*κ*B kinase activation [25] and inhibition of I*κ*B*α* degradation [20]. Also, Hsp72 induction by thermal pretreatment [21] attenuated proinflammatory cytokines [22] and improved survival in the LPS-induced systemic inflammation model, potentially involving Hsp-mediated inhibition of HMGB1 secretion [23]. A HS response induction of Hsp72 mRNA and protein expression in the lung has been shown to be associated with reduced lung injury [18], improved lung function [93], and survival [94].

Heat shock pretreatment could also attenuate the electrocortical dysfunction in rats with LPS-induced septic response, suggesting that HS induced Hsp72 might potentially be used to prevent septic encephalopathy in sepsis [24]. Similarly, HS treatment led to Hsp72 overexpression and preserved the expression of the enzyme mitochondrial cytochrome c oxidase complex associated with the minimization of ultrastructural deformities during sepsis [19]. Interestingly, Gln increased DNA binding of HSF1 in HS cells but in its absence ornithine was able to rescue the heat-induced DNA binding of HSF1 [43].

### 4.3. Human Studies

Although the release of the Hsp72 in sepsis serves as a host impending danger signal to neighboring cells and might exert a cytoprotective function at low serum levels, it might also potentiate an already active host immune response leading to poor outcome once a certain critical threshold is attained. Such a sensitive balance could be an explanation of the surprising finding of this study, showing that only 16.7% of the 6 human septic studies demonstrated an Hsp72 protective effect compared to 95.8% protection shown in the 41 septic animal studies. In addition, by experimentally studying healthy individuals rather than patients in a real clinical setting, human studies mix up mild molecular reactions to stress with severe infectious systemic inflammatory response syndrome (SIRS), being thereby unconvincing and unable to verify results of experimentally controlled septic animal models.

#### 4.3.1. Intracellular Hsp72: In Vitro Studies (Cell Models)

Human in vitro studies, mainly examining intracellular Hsp72 expression in hPBMC or hPMNL in patients and healthy individuals by using HS, Gln, exogenous Hsp72, and Hsp72 inhibitors or agonists, are inconclusive [57]. Thus, although Gln infusion altered neither endotoxin-induced systemic inflammation nor early expression of Hsp72 in isolated PBMCs in healthy volunteers [53], inducibility of ex vivo Hsp72 was impaired in peripheral blood lymphocytes of patients with severe sepsis [95], possibly contributing to immune dysfunction of T and B lymphocyte responses in resisting infection in severe sepsis [56].

Enhanced Hsp72 response in endurance-trained individuals, however, improved heat tolerance through both anti-inflammatory and antiapoptotic mechanisms [58]. Also, rHsp72 preconditioning ameliorated reactive oxygen species, TNF*α*, and CD11b/CD18 adhesion receptor expression after lipoteichoic acid addition [39]. Sepsis was shown to enhance expression of iHsp72 in PBMCs correlated to plasma TNF*α* concentrations [54] and in activated PMNLs, in which oxidative activity was increased and apoptosis was inhibited [55]. Similarly, using various Gln doses, proinflammatory cytokine release could directly be attenuated in PBMCs through enhancement of Hsp72 expression [61]. Overexpression of Hsp72 attenuated NF-*κ*B activation and proinflammatory cytokine release [88, 96], inhibited LPS-mediated apoptosis, and protected lung epithelial cells [80] and pulmonary artery endothelial cells from oxidant-mediated [97] and inflammation-induced lung injury [59].

#### 4.3.2. Extracellular Hsp72: In Vivo Studies (Serum Hsp)

Although PBMC Hsp72 expression was shown to be markedly decreased in critically ill septic patients [56], a significant increase in serum Hsp72 levels was reported in children with septic shock [52]. Extracellular Hsp72, reflected by increased serum levels, was also evident in children with acute lung injury [81] or following cardiopulmonary bypass [98]. Results of a recent adult study also indicated that increased serum Hsp72 is associated with mortality in sepsis [15]. Worse outcome associated with extracellular Hsp72 has also been reported in coronary artery disease [99], liver disease [90], sickle cell disease vasoocclusive crisis [100], and preeclampsia [101].

Heat shock proteins are markedly induced in response to a diverse range of cellular insults, being a reliable danger marker of cell stress [102]. Thus, extracellular Hsps act as a “danger signal,” activating immune-competent cells through LPS TLR4/CD14-dependent signaling [103]. According to the “danger hypothesis,” the release of stress proteins from severely stressed or damaged cells serves as a host impending danger signal to neighboring cells [104]. They are released in a nonspecific manner from dying, necrotic cells [105] or from viable cells release in a specific and inhibitable manner [106, 107]. Using viable cell counts and lactate dehydrogenase the release of Hsp72 was shown to not be due to cellular damage [60]. Recent studies suggest that Hsp72 is actively released via an exosome-dependent nonclassical protein secretory pathway, possibly involving lysosomal lipid rafts [108]. Immune cell receptors capture Hsps released from necrotic cells or Hsp-containing exosomes [109], and receptor engagement by Hsp72 increases dendritic cell production of  TNF*α*, IL-1b, IL-6, and chemokine [110]. The host innate immune response occurs through a NF-*κ*B-dependent proinflammatory gene expression via TLR4 and TLR2 [111], similar to a LPS-mediated signal transduction [112].

### 4.4. Factors Influencing Heat Shock Proteins Protective Role in Sepsis

Recent work demonstrated that febrile-range temperatures achieved during sepsis and noninfectious SIRS correlated with detectable changes in stress gene expression in vivo (whole blood messenger RNA), thereby suggesting that fever can activate Hsp72 gene expression and modify innate immune responses [113]. Hsp72 serum levels may also be modulated according to the patient oxidant status [15] and prevent excessive gut apoptosis and inflammation in an age-dependent response to sepsis [49]. Importantly, Hsp72 inhibited LPS-induced NO release but only partially reduced the LPS increased expression of iNOS mRNA and exhibited LPS-induced NF-*κ*B DNA binding and LPS tolerance; in contrast, HS inhibited LPS-induced NF-*κ*B and HSF1 activity whereas HSF1 inhibited NF-*κ*B DNA binding [45]!

A significant body of preexisting literature has hypothesized a relationship between Hsp72 expression and Gln's protection in both in vitro and in vivo settings [32, 43, 62, 114, 115]. Pioneer studies showed that Gln supplementation could attenuate lethal heat and oxidant injury and increase Hsp72 expression in intestinal epithelial cells [116–118]. Compared, however, with whey protein supplementation in a randomized, double-blinded, comparative effectiveness trial, zinc, selenium, Gln, and intravenous metoclopramide conferred no advantage in the immune-competent population [6]. In addition, we recently showed that although apparently safe in animal models (pups), premature infants, and critically ill children, glutamine supplementation did not reduce mortality or late onset sepsis [119]. Methodological problems noted in the reviewed randomized experimental and clinical trials [119] should therefore be seriously considered in any future well-designed large blinded randomized controlled trial involving glutamine supplementation in severe sepsis.

Drug interactions were also shown either to suppress Hsp72 protective effects exacerbating therefore drug-induced side effects or to induce Hsp72 beneficial effects by suppressing drug-induced exacerbations. Thus, it was recently shown that bleomycin-induced pulmonary fibrosis is mediated by suppression of pulmonary expression of Hsp72 whereas an inducer of Hsp72 expression, such as geranylgeranylacetone, could be therapeutically beneficial for the treatment of gefitinib-induced pulmonary fibrosis [120].

Finally, critically ill patients display variable physiologic responses when stressed; gene association studies have recently been employed to explain this variability. Genetic variants of Hsp72 have also been associated with the development of septic shock in patients [121, 122]. Thus, the specific absence of Hsp72.1/3 gene expression can lead to increased mortality after septic insult [85].

### 4.5. Limitations of the Study

The major problem that limits the comparability with human sepsis is the fact that in most cases of animal models, various forms of preconditioning were employed. This approach is nonspecific, and only a minor amount (about 10%) used genetically modified animals. Accordingly, important differences between cell and/or animal models versus clinical studies have been noted several times with various inflammatory pathways and have been written about extensively in the literature [123, 124]. To the best of our knowledge, however, such discrepancies have not been summarized in detail in the context of  Hsp72 and sepsis; in our opinion, these findings might be helpful for cautiously interpreting experimental data in the critical care field.

## 5. Conclusions

Heat shock proteins are molecular chaperokines that prevent the formation of nonspecific protein aggregates and exhibit sophisticated protection mechanisms. Experimental studies have repeatedly shown a strong molecular, biological, and clinical protective effect for Hsp72 in sepsis. Once again, clinical studies are inconclusive, varying from a protective in vitro effect to an in vivo Hsp72-mortality association. Possible influences by severity of disease-related factors, genetic variants, oxidant status, and unpredictable interventions such as those of temperature control, nutritional (glutamine) immune-enhancing, or drug intervening effects may unpredictably influence the Hsp72 protection efficacy in sepsis. Our “comparative” study data demonstrate that cell-protection with exogenous Hsp72, Hsp72 genes, heat stress, or glutamine is associated with induction of Hsp72 and that new Hsp72 targeted pharmaconutrition may be an approach to activating the preconditioning response in sepsis in clinical practice. However, as this hypothetical study suggests, much more work is needed to clarify the cellular and molecular mechanisms in which Hsp72 signals “danger” and regulates immune function in response to sepsis.

## Figures and Tables

**Table 1a:** (a)

In vivo	Induction	Organs studied	Expression in cells/Hsp72 challenge	Extracellular Hsp72 levels	Inhibitors	Functional	Pathways	Interleukins	Organ damage	Survival
CLP sepsis rats [18, 19] LPS-treated rats [20–24] LPS-treated mice [25]	Heat stress	Lungs (4) Heart (1) Splenocytes (1) Rostral Ventrolateral medulla (1) Mitochondrial function (1) Brain (1)	Induced (7)	—	Hsp70 inhibitors (KNK437 or pifithrin-m) abrogated the ability of the thermal treatment to enhance TNF-*α* (1)	Alleviated hypotension, bradycardia, sympathetic vasomotor activity (1) EEG and epileptic spikes attenuated (1)	Suppressed iNOS mRNA NF-*κ*B activation, IB kinase activation, IB degradation (1) Prevented downregulation of Grp75, preserved cytochrome c oxidase (1) enhanced phosphorylation of IKK, IkB, NF-*κ*B nuclear translocation, binding to the TNF-*α* promoter (1)	Cytokines declined (2) HMGB1 inhibited (1) enhanced LPS-induced TNF-*α* production (1)	Reduced (4) Prevented sepsis-associated encephalopathy (1)	Enhanced (6)

LPS-treated mice [26, 27] rats [28–30] sheep [31] CLP sepsis rats [32, 33] CPL sepsis mice [34, 35]	Glutamine	Heart (3) Lungs (3) Liver (2) Aorta (1) Kidneys (1) Brain (1) Blood (1) Multiple organs (1)	Induced (7)	Blood samples: increased Hsp72 only after coadministration of Gln and ciprofloxacin [26]	Quercetin blocked Gln-mediated enhancement of Hsp and HSF-1-p expressions and survival benefit (2) LD75 dose of *P. aeruginosa* and ciprofloxacin in combinations (1)	Prevented ARDS (2) arterial pressure, cardiac contractility restored in the Gln than in the LPS shock (2) Quercetin prevented Gln protection (1) No difference in hemodynamic parameters (1)	Inhibited activation, translocation of NF-*κ*B to the nucleus degradation of IKBalpha, phosphorylation of p38 MAPK, ERK, increased MKP-1 (1) lung HMGB-1 expression NF-*κ*B DNA-binding activity suppressed (1) Reduced peroxide biosynthesis (1)	Attenuated TNF-alpha (3), IL-6. IL-18, MDA, HMGB-1, apoptosis (1) increased IL-10 (1)	Reduced (5)	Enhanced (7)
LPS-treated rats bovine or Ad70 virus or rHsp [36–40] CLP sepsis rats and tracheas AdHSP [32]	Exogenous rHsp AdTrack or Ad70 virus 72	Liver (1) Peritoneal macrophages (1) MLE-12 cells (1) Myocardium (1) Lungs (1)	Induced (4)	—	—	Normalized hemostasis (2) hemodynamics (2) Biochemical parameters (1)	Inhibited LPS-induced decrease NO expression in macrophages, normalized neutrophil apoptosis (1) inhibited I*κ*B*α* degradation and NF-*κ*B, p65 nuclear translocation (2) apoptotic cellular pathways caspases 3, 8, 9 (1)	Modified myeloid cells response to LPS (1) prevented LPS-induced increase in TNF-*α* and IL-6 (2) Reduced ICAM-1, attenuated cardiac dysfunction (1)	Attenuated cardiac dysfunction (1) reduced alveolar cell apoptosis (1)	Enhanced (5)

**Table 1b:** (b)

In vitro	Induction	Organs studied	Intracellular Hsp72 expression	Inhibitors	Pathways	Interleukins	Organ damage	Survival
Murine macrophage-like RAW 264.7 cells [12]	Heat shocked	Macrophages (1)	Cells from HS overexpressed Hsp72 (1)	—	Inhibited phosphorylation of p38, JNK, ERK/MAPK, I*κ*B*α* degradation, NF-*κ*B p65 nuclear translocation (1)	HS inhibited HMGB1-induced cytokines TNF-*α* and IL-1*β* (1)	—	Enhanced (1)

CLP-treated murine peritoneal macrophage cell line RAW264.7 [41] Neonatal rat cardiomyocytes [42] IEC-18 rat intestinal epithelial cells [43]	Glutamine	Peritoneal macrophages (1) Cardiomyocytes (1) Intestinal epithelial cells (1)	Increased Hsp70 expression (2)	Gln protection mimicked by PUGNA, banished by alloxan (1) DFMO ornithine decarboxylase inhibitor (1)	Reduced LDH, increased O-ClcNAc, HSF-1, transcription activity (1) increased HSF1 binding to HSE (1)	In vitro TNF-*α* dose- time-Gln. Dependent In vivo lower intracellular TNF-*α* level	Attenuated LPS-induced cardiomyocyte damage (1)	Enhanced (3)

LPS-treated rats [44] LPS stimulation-mouse macrophage-like cell line (RAW 264.7 cells) [45, 46]	Transfected with Hsp70 plasmid or HS	Myocardium (1) Macrophages (2)	Hsp70 plasmid or HS induced Hsp70 (2)	—	iNOS mRNA completely abolished by HS-Hsp70–transfected cells (1) HS inhibited LPS-induced NF-*κ*B and HSF-1 activity (1) increases both cellular SK1 mRNA and protein levels (1)	—	—	Enhanced (2)

CLP rats, murine lung epithelial-12 cells in culture [47] Murine macrophage-like RAW 264.7 cells [12]	Exogenous Hsp72	Lungs (1) Macrophages (1)	Overexpression of Hsp72 in RAW/Hsp72 cells (1) —	—	Limited nuclear translocation of NF-*κ*B, phosphorylation of IkappaBalpha (2) Inhibition of the MAP kinases (p38, JNK, and ERK) (1)	Inhibition of the NF-*κ*B - HMGB1-induced release of TNF-*α*, IL-1*β* (1)	Limited NF-*κ*B activation (2)	Enhanced (2)

CLP-treated mice [48]	Arsenite (Positive control)	Lungs (1)	Induced- Inhibitors blocked Hsp72 expression, (1)	Anti-human Hsp72 (1)	Pretreatment with neutralizing antibodies to Hsp72 diminished neutrophil killing (1)	—	Survivors higher *n* of *γδ*T cells (1)	Enhanced (1)

**Table 1c:** (c)

KO animals	Induction	Organs studied	Intracellular Hsp72 expression	Pathways	Interleukins	Organ damage	Survival
CLP sepsis Hsp70.1/3−/− KO mice [34]	Glutamine	Lungs (1)	Hsp70.1/3−/− mice did not increase Hsp72 (1)	Hsp70.1/3((−/−)) mice increased NF-*κ*B binding/ activation (1)	Increased TNF-*α*, IL-6 in KO (1)	Increased lung injury in KO (1)	Decreased in KO (1)

CLP sepsis, injection of microorganisms Hsp70−/− KO mice [49]	Imipenem/ cilastatin	Gut (1) Lungs (1)	Hsp70−/−mice did not increase Hsp72 (1)	Increased apoptosis and inflammation	Hsp70−/−increased TNF-*α*, IL-6, IL-10, IL-1b	KO-increased gut epithelial apoptosis, pulmonary inflammation (1)	Decreased in KO age dependent (1)

LPS-treated mice Hsp−/− or overexpressed Hsp70 [50]	LPS	Intestinal epithelium (1)	Pharmacologic Hsp70 upregulation	Hsp70 reduced TLR4 signaling in enterocytes (1)	Hsp70 reversed TLR4- cytokines, enterocyte apoptosis (1)	Prevented and treated experimental NEC (1)	—

LPS-treated mice overexpressing the human Hspa12b gene [51]	LPS	Heart (1)	Overexpression of HSPA12B	Prevented decrement in the activation of PI3K/protein kinase B signaling in myocardium (1)	Decreased the expression of VCAM-1/ICAM-1 (1)	Decreased leucocyte infiltration in myocardium (1) Attenuated cardiac dysfunction (1)	—

*n*: number of studies; PBMC: peripheral blood mononuclear cells; LPS: bacterial lipopolysaccharide; CLP: cecal ligation and puncture; TNF-*α*: tumor necrosis factor-alpha; AdHSP: adenoviral vector Hsp72; Gln: glutamine; HS: heat stress; Hspgene: Hsp70 gene-transfected models; HSF1: HS factor 1; HSE: heat shock element; IKK: I*κ*B kinase; IkB: IkappaBalpha.

**Table 2a:** (a)

In vivo	Study population/material	Expression in cells/Hsp72 challenge	Extracellular Hsp72 levels	Hsp72 is associated with	Conclusion on the Hsp72 role in sepsis
Patients with septic shock [15, 52]	Children with septic shock (1), adults with severe sepsis (1)	—	Elevated in septic shock (1) nonsurvivors (1) pronounced oxidative damage (1)	Septic shock-mortality (2) modulated according to oxidant status (1)	Related to mortality (2) patient oxidant status (1)

Healthy young men Gln-LPS [53]	Crossover study: Hsp70 in PBMCs (1)	Gln did not affect Hsp70 in PBMCs (1)	—	Gln did not affect LPS-WBC, TBF-*α*, IL-6, temperature heart rate alterations	Not protective in experimental sepsis (1)

**Table 2b:** (b)

In vitro	Study population/material	Expression in cells/Hsp72 challenge	Hsp70 is associated with	Conclusion on the Hsp72 role in sepsis
PBMCs-Hsp inhibitor-inducers [54, 55]	PBMCs 24 hours after sepsis (1) sodium arsenite (inducer of Hsp) and quercetin (suppressor of Hsp) to regulate expression of Hsp70 in PMNLs (1)	Hsp70 increased (1) prevented by quercetin (1)	Enhanced TNF-*α* (1) increased oxidative activity, inhibited apoptosis (1)	Inconclusive (1) may inhibit apoptosis (1)

LPS-PBMC [56]	LPS inducibility of Hsp70 expression in the PBMC	Inhibits Hsp70 expression in PBMC (in septic patients more than in controls)	Decreased resistance to infectious insults during severe sepsis	May be related to infections

Heat shock, PBMC [57–60]	Heat stress Hsp70 in PBMC (2) or with LPS and training (1) or exercised in heat acclimation (1)	Hsp70 increase (3) inhibited by monensin, methyl-beta-cyclodextrin, and methylamine, reduced in patients with ARDS (2)	Hsp70 decreased in ARDS, recover *εδ* over time (1) released from lysosomal lipid rafts (1) Reduced apoptosis, TNF-*α*, IL-1b, increase *δ* CD14/CD16 (1)	Protective (3) not sufficient (1)

Recombinant Hsp70-neutrophils, monocytes [39]	Preconditioning of myeloid cells after LTA addition with rHsp70 (1)	Effect of human recombinant Hsp70 isolated from *Spodoptera* cells on neutrophil apoptosis and expression of CD11b/CD18 receptors and TNF on ROS production in neutrophils and monocytes	Ameliorated reactive oxygen species, TNF-*α*, CD11b/CD18, did not normalize apoptosis (1)	Protective (1)

Glutamine-[61]-lymphocytes [62]	Glutamine-PBMCs (1) or lymphocytes (1)	After LPS-HS increased 3-fold Hsp70. A reduction of Gln led to a 40% lower Hsp70 level (2)	Gln decreased TNF-*α* (1) Reduced Gln = reduced Hsp70 = impaired stress response (1)	Protective (2)

## References

[1] Dal-Pizzol F (2004). Alternative activated macrophage: a new key for systemic inflammatory response syndrome and sepsis treatment?. *Critical Care Medicine*.

[2] Sadique MZ, Grieve R, Harrison DA, Cuthbertson BH, Rowan KM (2011). Is Drotrecogin alfa (activated) for adults with severe sepsis, cost-effective in routine clinical practice?. *Critical Care*.

[3] Carcillo J, Holubkov R, Dean JM (2009). Rationale and design of the pediatric critical illness stress-induced immune suppression (CRISIS) prevention trial. *Journal of Parenteral and Enteral Nutrition*.

[4] Dare AJ, Phillips ARJ, Hickey AJR (2009). A systematic review of experimental treatments for mitochondrial dysfunction in sepsis and multiple organ dysfunction syndrome. *Free Radical Biology and Medicine*.

[5] Ranieri VM, Thompson BT, Barie PS (2012). Drotrecogin alfa (activated) in adults with septic shock. *The New England Journal of Medicine*.

[6] Carcillo JA, Dean JM, Holubkov R (2012). The randomized comparative pediatric critical illness stress-induced immune suppression (CRISIS) prevention trial. *Pediatric Critical Care Medicine*.

[7] Silver JT, Noble EG (2012). Regulation of survival gene hsp70. *Cell stress & chaperones*.

[8] Jiang B, Liang P, Deng G, Tu Z, Liu M, Xiao X (2011). Increased stability of Bcl-2 in HSP70-mediated protection against apoptosis induced by oxidative stress. *Cell Stress and Chaperones*.

[9] Lee BH, Inui D, Suh GY (2012). Association of body temperature and antipyretic treatments with mortality of critically ill patients with and without sepsis: multi-centered prospective observational study. *Critical Care*.

[10] Schortgen F, Clabault K, Katsahian S (2012). Fever control using external cooling in septic shock: a randomized controlled trial. *American Journal of Respiratory and Critical Care Medicine*.

[11] Johnson JD, Fleshner M (2006). Releasing signals, secretory pathways, and immune function of endogenous extracellular heat shock protein 72. *Journal of Leukocyte Biology*.

[12] Tang D, Kang R, Xiao W, Wang H, Calderwood SK, Xiao X (2007). The anti-inflammatory effects of heat shock protein 72 involve inhibition of high-mobility-group box 1 release and proinflammatory function in macrophages. *Journal of Immunology*.

[13] Calderwood SK, Mambula SS, Gray PJ, Theriault JR (2007). Extracellular heat shock proteins in cell signaling. *FEBS Letters*.

[14] Calderwood SK, Gong J (2012). Molecular chaperones in mammary cancer growth and breast tumor therapy. *Journal of Cellular Biochemistry*.

[15] Gelain DP, De Bittencourt Pasquali MA, M. Comim C (2011). Serum heat shock protein 70 levels, oxidant status, and mortality in sepsis. *Shock*.

[16] Xu Q, Metzler B, Jahangiri M, Mandal K (2012). Molecular chaperones and heat shock proteins in atherosclerosis. *American Journal of Physiology*.

[17] Moher D, Liberati A, Tetzlaff J, Altman DG (2009). Preferred reporting items for systematic reviews and meta-analyses: the PRISMA statement. *Journal of Clinical Epidemiology*.

[18] Villar J, Ribeiro SP, Mullen JBM, Kuliszewski M, Post M, Slutsky AS (1994). Induction of the heat shock response reduces mortality rate and organ damage in a sepsis-induced acute lung injury model. *Critical Care Medicine*.

[19] Chen H-W, Kuo H-T, Lu T-S, Wang S-J, Yang R-C (2004). Cytochrome c oxidase as the target of the heat shock protective effect in septic liver. *International Journal of Experimental Pathology*.

[20] Chan JYH, Ou C-C, Wang L-L, Chan SHH (2004). Heat shock protein 70 confers cardiovascular protection during endotoxemia via inhibition of nuclear factor-*κ*B activation and inducible nitric oxide synthase expression in the rostral ventrolateral medulla. *Circulation*.

[21] Ofenstein JP, Heidemann S, Juett-Wilstermann A, Sarnaik A (2000). Expression of stress proteins HSP 72 and HSP 32 in response to endotoxemia. *Annals of Clinical and Laboratory Science*.

[22] Chu EK, Ribeiro SP, Slutsky AS (1997). Heat stress increases survival rates in lipopolysaccharide-stimulated rats. *Critical Care Medicine*.

[23] Hasegawa A, Iwasaka H, Hagiwara S, Noguchi T (2011). Relationship between HMGB1 and tissue protective effects of HSP72 in a LPS-induced systemic inflammation model. *Journal of Surgical Research*.

[24] Lin L-C, Chen Y-Y, Lee W-T, Chen H-L, Yang R-C (2010). Heat shock pretreatment attenuates sepsis-associated encephalopathy in LPS-induced septic rats. *Brain and Development*.

[25] Lee C-T, Zhong L, Mace TA, Repasky EA (2012). Elevation in body temperature to fever range enhances and prolongs subsequent responsiveness of macrophages to endotoxin challenge. *PLoS ONE*.

[26] Mazloomi E, Jazani NH, Sohrabpour M, Ilkhanizadeh B, Shahabi S (2011). Synergistic effects of glutamine and ciprofloxacin in reduction of Pseudomonas aeruginosa-induced septic shock severity. *International Immunopharmacology*.

[27] Chen G, Neilan TG, Chen H (2010). Attenuation of lipopolysaccharide-mediated left ventricular dysfunction by glutamine preconditioning. *Journal of Surgical Research*.

[28] Wischmeyer PE, Kahana M, Wolfson R, Ren H, Musch MM, Chang EB (2001). Glutamine induces heat shock protein and protects against endotoxin shock in the rat. *Journal of Applied Physiology*.

[29] Jing L, Wu Q, Wang F (2007). Glutamine induces heat-shock protein and protects against Escherichia coli lipopolysaccharide-induced vascular hyporeactivity in rats. *Critical Care*.

[30] Zhao YJ, Wang H, Liu X, Sun M, Kazuhiro H (2012). Protective effects of glutamine in a rat model of endotoxemia. *Molecular Medicine Reports*.

[31] Scharte M, Baba HA, Van Aken H (2001). Alanyl-glutamine dipeptide does not affect hemodynamics despite a greater increase in myocardial heat shock protein 72 immunoreactivity in endotoxemic sheep. *Journal of Nutrition*.

[32] Singleton KD, Serkova N, Beckey VE, Wischmeyer PE (2005). Glutamine attenuates lung injury and improves survival after sepsis: role of enhanced heat shock protein expression. *Critical Care Medicine*.

[33] Kwon WY, Suh GJ, Kim KS (2010). Glutamine attenuates acute lung injury by inhibition of high mobility group box protein-1 expression during sepsis. *British Journal of Nutrition*.

[34] Singleton KD, Wischmeyer PE (2007). Glutamine’s protection against sepsis and lung injury is dependent on heat shock protein 70 expression. *American Journal of Physiology*.

[35] Hu Y-M, Pai M-H, Yeh C-L, Hou Y-C, Yeh S-L (2012). Glutamine administration ameliorates sepsis-induced kidney injury by downregulating the high-mobility group box protein-1-mediated pathway in mice. *American Journal of Physiology*.

[36] Dokladny K, Lobb R, Wharton W, Ma TY, Moseley PL (2010). LPS-induced cytokine levels are repressed by elevated expression of HSP70 in rats: possible role of NF-*κ*B. *Cell Stress and Chaperones*.

[37] Rozhkova E, Yurinskaya M, Zatsepina O (2010). Exogenous mammalian extracellular HSP70 reduces endotoxin manifestations at the cellular and organism levels. *Annals of the New York Academy of Sciences*.

[38] Su X, Sykes JB, Ao L, Raeburn CD, Fullerton DA, Meng X (2010). Extracellular heat shock cognate protein 70 induces cardiac functional tolerance to endotoxin: differential effect on TNF-*α* and ICAM-1 levels in heart tissue. *Cytokine*.

[39] Vinokurov M, Ostrov V, Yurinskaya M (2012). Recombinant human Hsp70 protects against lipoteichoic acid-induced inflammation manifestations at the cellular and organismal levels. *Cell Stress & Chaperones*.

[40] Kustanova GA, Murashev AN, Karpov VL (2006). Exogenous heat shock protein 70 mediates sepsis manifestations and decreases the mortality rate in rats. *Cell Stress and Chaperones*.

[41] Liang M, Wang X, Yuan Y, Zhou Q, Tong C, Jiang W (2009). Different effect of glutamine on macrophage tumor necrosis factor-alpha release and heat shock protein 72 expression in vitro and in vivo. *Acta Biochimica et Biophysica Sinica*.

[42] Gong J, Jing L (2011). Glutamine induces heat shock protein 70 expression via O-GlcNAc modification and subsequent increased expression and transcriptional activity of heat shock factor-1. *Minerva Anestesiologica*.

[43] Iwashita Y, Sakiyama T, Musch MW, Ropeleski MJ, Tsubouchi H, Chang EB (2011). Polyamines mediate glutamine-dependent induction of the intestinal epithelial heat shock response. *American Journal of Physiology*.

[44] Meldrum DR, Meng X, Shames BD (1999). Liposomal delivery of heat-shock protein 72 into the heart prevents endotoxin-induced myocardial contractile dysfunction. *Surgery*.

[45] Song M, Pinsky MR, Kellum JA (2008). Heat shock factor 1 inhibits nuclear factor-*κ*B nuclear binding activity during endotoxin tolerance and heat shock. *Journal of Critical Care*.

[46] Ding XZ, Feng XR, Borschel RH (2010). HSP-70 mitigates LPS/SKI-induced cell damage by increasing sphingosine kinase 1 (SK1). *Prostaglandins and Other Lipid Mediators*.

[47] Weiss YG, Bromberg Z, Raj N (2007). Enhanced heat shock protein 70 expression alters proteasomal degradation of I*κ*B kinase in experimental acute respiratory distress syndrome. *Critical Care Medicine*.

[48] Hirsh MI, Hashiguchi N, Chen Y, Yip L, Junger WG (2006). Surface expression of HSP72 by LPS-stimulated neutrophils facilitates *γδ*T cell-mediated killing. *European Journal of Immunology*.

[49] McConnell KW, Fox AC, Clark AT (2011). The role of heat shock protein 70 in mediating age-dependent mortality in sepsis. *Journal of Immunology*.

[50] Afrazi A, Sodhi CP, Good M (2012). Intracellular heat shock protein-70 negatively regulates TLR4 signaling in the newborn intestinal epithelium. *Journal of Immunology*.

[51] Zhou H, Qian J, Li C (2011). Attenuation of cardiac dysfunction by HSPA12B in endotoxin-induced sepsis in mice through a PI3K-dependent mechanism. *Cardiovascular Research*.

[52] Wheeler DS, Fisher LE, Catravas JD, Jacobs BR, Carcillo JA, Wong HR (2005). Extracellular hsp70 levels in children with septic shock. *Pediatric Critical Care Medicine*.

[53] Andreasen AS, Pedersen-Skovsgaard T, Mortensen OH, Van Hall G, Moseley PL, Pedersen BK (2009). The effect of glutamine infusion on the inflammatory response and HSP70 during human experimental endotoxaemia. *Critical Care*.

[54] Delogu G, Lo Bosco L, Marandola M (1997). Heat shock protein (HSP70) expression in septic patients. *Journal of Critical Care*.

[55] Hashiguchi N, Ogura H, Tanaka H (2001). Enhanced expression of heat shock proteins in activated polymorphonuclear leukocytes in patients with sepsis. *Journal of Trauma*.

[56] Schroeder S, Bischoff J, Lehmann LE (1999). Endotoxin inhibits heat shock protein 70 (HSP7O) expression in peripheral blood mononuclear cells of patients with severe sepsis. *Intensive Care Medicine*.

[57] Amorim F, Yamada P, Robergs R, Schneider S, Moseley P (2011). Effects of whole-body heat acclimation on cell injury and cytokine responses in peripheral blood mononuclear cells. *European Journal of Applied Physiology*.

[58] Selkirk GA, McLellan TM, Wright HE, Rhind SG (2009). Expression of intracellular cytokines, HSP72, and apoptosis in monocyte subsets during exertional heat stress in trained and untrained individuals. *American Journal of Physiology*.

[59] Durand P, Bachelet M, Brunet F (2000). Inducibility of the 70 kD heat shock protein in peripheral blood monocytes is decreased in human acute respiratory distress syndrome and recovers over time. *American Journal of Respiratory and Critical Care Medicine*.

[60] Hunter-Lavin C, Davies EL, Bacelar MMFVG, Marshall MJ, Andrew SM, Williams JHH (2004). Hsp70 release from peripheral blood mononuclear cells. *Biochemical and Biophysical Research Communications*.

[61] Wischmeyer PE, Riehm J, Singleton KD (2003). Glutamine attenuates tumor necrosis factor-*α* release and enhances heat shock protein 72 in human peripheral blood mononuclear cells. *Nutrition*.

[62] Oehler R, Pusch E, Dungel P (2002). Glutamine depletion impairs cellular stress response in human leucocytes. *British Journal of Nutrition*.

[63] Pockley AG, Muthana M, Calderwood SK (2008). The dual immunoregulatory roles of stress proteins. *Trends in Biochemical Sciences*.

[64] Voellmy R (2004). On mechanisms that control heat shock transcription factor activity in metazoan cells. *Cell Stress and Chaperones*.

[65] Shi Y, Mosser DD, Morimoto RI (1998). Molecular chaperones as HSF1-specific transcriptional repressors. *Genes and Development*.

[66] Amin J, Ananthan J, Voellmy R (1988). Key features of heat shock regulatory elements. *Molecular and Cellular Biology*.

[67] Pirkkala L, Nykänen P, Sistonen L (2001). Roles of the heat shock transcription factors in regulation of the heat shock response and beyond. *FASEB Journal*.

[68] De Maio A (1999). Heat shock proteins: facts, thoughts, and dreams. *Shock*.

[69] Trotter EW, Kao CM-F, Berenfeld L, Botstein D, Petsko GA, Gray JV (2002). Misfolded proteins are competent to mediate a subset of the responses to heat shock in Saccharomyces cerevisiae. *Journal of Biological Chemistry*.

[70] Voellmy R (1995). Transduction of the stress signal and mechanisms of transcriptional regulation of heat shock/stress protein gene expression in higher eukaryotes. *Critical Reviews in Eukaryotic Gene Expression*.

[71] Radsak MP, Hilf N, Singh-Jasuja H (2003). The heat shock protein Gp96 binds to human neutrophils and monocytes and stimulates effector functions. *Blood*.

[72] Pockley AG (2003). Heat shock proteins as regulators of the immune response. *Lancet*.

[73] Robert J (2003). Evolution of heat shock protein and immunity. *Developmental and Comparative Immunology*.

[74] Kregel KC (2002). Invited review: heat shock proteins: modifying factors in physiological stress responses and acquired thermotolerance. *Journal of Applied Physiology*.

[75] Lai Y, Kochanek PM, Adelson PD, Janesko K, Ruppel RA, Clark RSB (2004). Induction of the stress response after inflicted and non-inflicted traumatic brain injury in infants and children. *Journal of Neurotrauma*.

[76] Gupta S, Deepti A, Deegan S, Lisbona F, Hetz C, Samali A (2010). HSP72 protects cells from ER stress-induced apoptosis via enhancement of IRE1*α*-xbp1 signaling through a physical interaction. *PLoS Biology*.

[77] Li GC, Li L, Liu Y-K, Mak JY, Chen L, Lee WMF (1991). Thermal response of rat fibroblasts stably transfected with the human 70-kDa heat shock protein-encoding gene. *Proceedings of the National Academy of Sciences of the United States of America*.

[78] Wong HR, Mannix RJ, Rusnak JM (1996). The Heat-shock response attenuates lipopolysaccharide-mediated apoptosis in cultured sheep pulmonary artery endothelial cells. *American Journal of Respiratory Cell and Molecular Biology*.

[79] Wong HR, Ryan M, Menendez IY, Denenberg A, Wispé JR (1997). Heat shock protein induction protects human respiratory epithelium against nitric oxide-mediated cytotoxicity. *Shock*.

[80] Wong HR, Menendez IY, Ryan MA, Denenberg AG, Wispé JR (1998). Increased expression of heat shock protein-70 protects A549 cells against hyperoxia. *American Journal of Physiology*.

[81] Wheeler DS, Wong HR (2007). Heat shock response and acute lung injury. *Free Radical Biology and Medicine*.

[82] Hiratsuka M, Mora BN, Yano M, Mohanakumar T, Patterson GA (1999). Gene transfer of heat shock protein 70 protects lung grafts from ischemia-reperfusion injury. *Annals of Thoracic Surgery*.

[83] Riabowol KT, Mizzen LA, Welch WJ (1988). Heat shock is lethal to fibroblasts microinjected with antibodies against hsp70. *Science*.

[84] McMillan DR, Xiao X, Shao L, Graves K, Benjamin IJ (1998). Targeted disruption of heat shock transcription factor 1 abolishes thermotolerance and protection against heat-inducible apoptosis. *Journal of Biological Chemistry*.

[85] Singleton KD, Wischmeyer PE (2006). Effects of HSP70.1/3 gene knockout on acute respiratory distress syndrome and the inflammatory response following sepsis. *American Journal of Physiology*.

[86] Weiss YG, Maloyan A, Tazelaar J, Raj N, Deutschman CS (2002). Adenoviral transfer of HSP-70 into pulmonary epithelium ameliorates experimental acute respiratory distress syndrome. *Journal of Clinical Investigation*.

[87] Aschkenasy G, Bromberg Z, Raj N, Deutschman CS, Weiss YG (2011). Enhanced Hsp70 expression protects against acute lung injury by modulating apoptotic pathways. *PLoS ONE*.

[88] Weiss YG, Bouwman A, Gehan B, Schears G, Raj N, Deutschman CS (2000). Cecal ligation and double puncture impairs heat shock protein 70 (HSP-70) expression in the lungs of rats. *Shock*.

[89] Bromberg Z, Raj N, Goloubinoff P, Deutschman CS, Weiss YG (2008). Enhanced expression of 70-kilodalton heat shock protein limits cell division in a sepsis-induced model of acute respiratory distress syndrome. *Critical Care Medicine*.

[90] Kimura F, Itoh H, Ambiru S (2004). Circulating heat-shock protein 70 is associated with postoperative infection and organ dysfunction after liver resection. *American Journal of Surgery*.

[91] Wischmeyer PE, Kahana M, Wolfson R, Ren H, Musch MM, Chang EB (2001). Glutamine reduces cytokine release, organ damage, and mortality in a rat model of endotoxemia. *Shock*.

[92] Singleton KD, Beckey VE, Wischmeyer PE (2005). Glutamine prevents activation of NF-*κ*B and stress kinase pathways, attenuates inflammatory cytokine release, and prevents acute respiratory distress syndrome (ARDS) following sepsis. *Shock*.

[93] Ribeiro SP, Rhee K, Tremblay L, Veldhuizen R, Lewis JF, Slutsky AS (2001). Heat stress attenuates ventilator-induced lung dysfunction in an ex vivo rat lung model. *American Journal of Respiratory and Critical Care Medicine*.

[94] Ribeiro SP, Villar J, Downey GP, Edelson JD, Slutsky AS (1994). Sodium arsenite induces heat shock protein-72 kilodalton expression in the lungs and protects rats against sepsis. *Critical Care Medicine*.

[95] Schroeder S, Lindemann C, Hoeft A (1999). Impaired inducibility of heat shock protein 70 in peripheral blood lymphocytes of patients with severe sepsis. *Critical Care Medicine*.

[96] Feinstein DL, Galea E, Aquino DA, Li GC, Xu H, Reis DJ (1996). Heat shock protein 70 suppresses astroglial-inducible nitric-oxide synthase expression by decreasing NF*κ*B activation. *Journal of Biological Chemistry*.

[97] Wong HR, Ryan M, Gebb S, Wispé JR (1997). Selective and transient in vitro effects of heat shock on alveolar type II cell gene expression. *American Journal of Physiology*.

[98] Dybdahl B, Wahba A, Haaverstad R (2004). On-pump versus off-pump coronary artery bypass grafting: more heat-shock protein 70 is released after on-pump surgery. *European Journal of Cardio-thoracic Surgery*.

[99] Dybdahl B, Slørdahl SA, Waage A, Kierulf P, Espevik T, Sundan A (2005). Myocardial ischaemia and the inflammatory response: release of heat shock protein 70 after myocardial infarction. *Heart*.

[100] Adewoye AH, Klings ES, Farber HW (2005). Sickle cell vaso-occlusive crisis induces the release of circulating serum heat shock protein-70. *American Journal of Hematology*.

[101] Fukushima A, Kawahara H, Isurugi C (2005). Changes in serum levels of heat shock protein 70 in preterm delivery and pre-eclampsia. *Journal of Obstetrics and Gynaecology Research*.

[102] Oberbeck R, Deckert H, Bangen J, Kobbe P, Schmitz D (2007). Dehydroepiandrosterone: a modulator of cellular immunity and heat shock protein 70 production during polymicrobial sepsis. *Intensive Care Medicine*.

[103] Aneja R, Odoms K, Dunsmore K, Shanley TP, Wong HR (2006). Extracellular heat shock protein-70 induces endotoxin tolerance in THP-1 cells. *Journal of Immunology*.

[104] Lang A, Benke D, Eitner F (2005). Heat shock protein 60 is released in immune-mediated glomerulonephritis and aggravates disease: in vivo evidence for an immunologic danger signal. *Journal of the American Society of Nephrology*.

[105] Basu S, Binder RJ, Suto R, Anderson KM, Srivastava PK (2000). Necrotic but not apoptotic cell death releases heat shock proteins, which deliver a partial maturation signal to dendritic cells and activate the NF-*κ*B pathway. *International Immunology*.

[106] Lancaster GI, Møller K, Nielsen B, Secher NH, Febbraio MA, Nybo L (2004). Exercise induces the release of heat shock protein 72 from the human brain in vivo. *Cell Stress and Chaperones*.

[107] Guzhova I, Kislyakova K, Moskaliova O (2001). In vitro studies show that Hsp70 can be released by glia and that exogenous Hsp70 can enhance neuronal stress tolerance. *Brain Research*.

[108] Lancaster GI, Febbraio MA (2005). Exosome-dependent trafficking of HSP70: a novel secretory pathway for cellular stress proteins. *Journal of Biological Chemistry*.

[109] Clayton A, Turkes A, Navabi H, Mason MD, Tabi Z (2005). Induction of heat shock proteins in B-cell exosomes. *Journal of Cell Science*.

[110] Asea A, Kraeft S-K, Kurt-Jones EA (2000). HSP70 stimulates cytokine production through a CD 14-dependant pathway, demonstrating its dual role as a chaperone and cytokine. *Nature Medicine*.

[111] Asea A, Rehli M, Kabingu E (2002). Novel signal transduction pathway utilized by extracellular HSP70. Role of toll-like receptor (TLR) 2 and TLR4. *Journal of Biological Chemistry*.

[112] Anderson KM, Srivastava PK (2000). Heat, heat shock, heat shock proteins and death: a central link in innate and adaptive immune responses. *Immunology Letters*.

[113] Sonna LA, Hawkins L, Lissauer ME (2010). Core temperature correlates with expression of selected stress and immunomodulatory genes in febrile patients with sepsis and noninfectious SIRS. *Cell stress & chaperones*.

[114] Ziegler TR, Ogden LG, Singleton KD (2005). Parenteral glutamine increases serum heat shock protein 70 in critically ill patients. *Intensive Care Medicine*.

[115] Grau T, Bonet A, Miñambres E (2011). The effect of l-alanyl-l-glutamine dipeptide supplemented total parenteral nutrition on infectious morbidity and insulin sensitivity in critically ill patients. *Critical Care Medicine*.

[116] Wischmeyer PE, Musch MW, Madonna MB, Thisted R, Chang EB (1997). Glutamine protects intestinal epithelial cells: role of inducible HSP70. *American Journal of Physiology*.

[117] Musch MW, Hayden D, Sugi K, Straus D, Chang EB (1998). Cell-specific induction of hsp72-mediated protection by glutamine against oxidant injury in IEC18 cells. *Proceedings of the Association of American Physicians*.

[118] Chow A, Zhang R (1998). Glutamine reduces heat shock-induced cell death in rat intestinal epithelial cells. *Journal of Nutrition*.

[119] Briassouli E, Briassoulis G (2012). Glutamine randomized studies in early life: the unsolved riddle of experimental and clinical studies. *Clinical and Developmental Immunology*.

[120] Namba T, Tanaka K-I, Hoshino T, Azuma A, Mizushima T (2011). Suppression of expression of heat shock protein 70 by gefitinib and its contribution to pulmonary fibrosis. *PLoS ONE*.

[121] Kee C, Cheong KY, Pham K, Waterer GW, Temple SEL (2008). Genetic variation in heat shock protein 70 is associated with septic shock: narrowing the association to a specific haplotype. *International Journal of Immunogenetics*.

[122] Waterer GW, ElBahlawan L, Quasney MW, Zhang Q, Kessler LA, Wunderink RG (2003). Heat shock protein 70-2 + 1267 AA homozygotes have an increased risk of septic shock in adults with community-acquired pneumonia. *Critical Care Medicine*.

[123] Shirey KA, Lai W, Scott AJ (2013). The TLR4 antagonist Eritoran protects mice from lethal influenza infection. *Nature*.

[124] Opal SM, Laterre PF, Francois B (2013). Effect of eritoran, an antagonist of MD2-TLR4, on mortality in patients with severe sepsis: the ACCESS randomized trial. *JAMA*.

